# Supplementary figures and images for: Parent of origin gene expression in a founder population identifies two new candidate imprinted genes at known imprinted regions
Source: PLoS One. 2018 Sep 11;13(9):e0203906. doi: 10.1371/journal.pone.0203906 (PMC6133383; doi:10.1371/journal.pone.0203906)

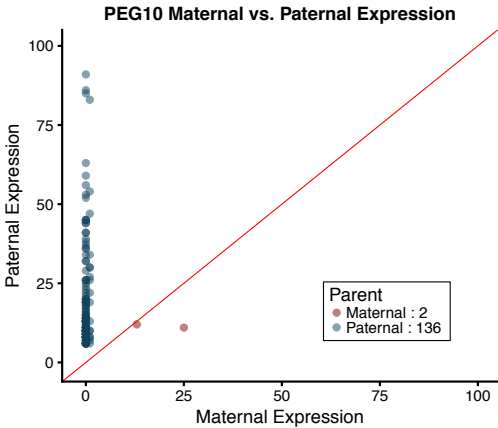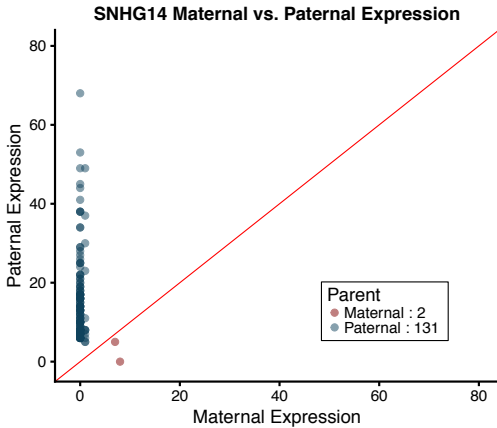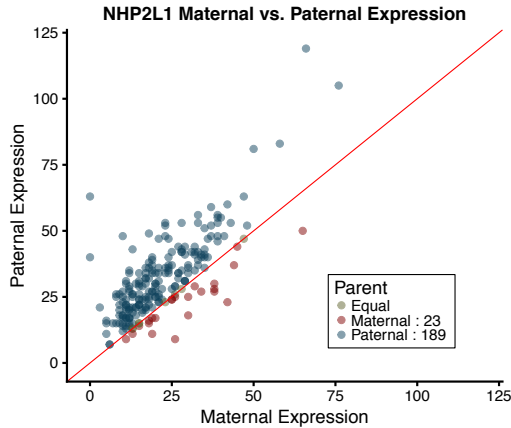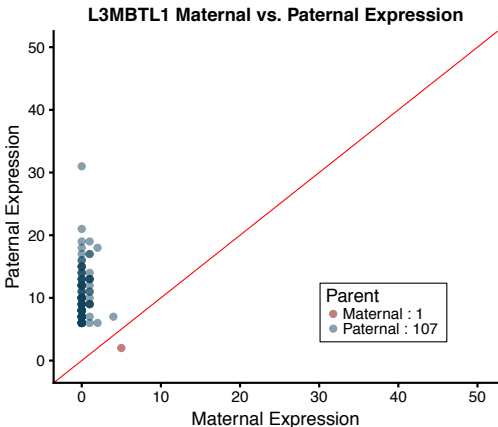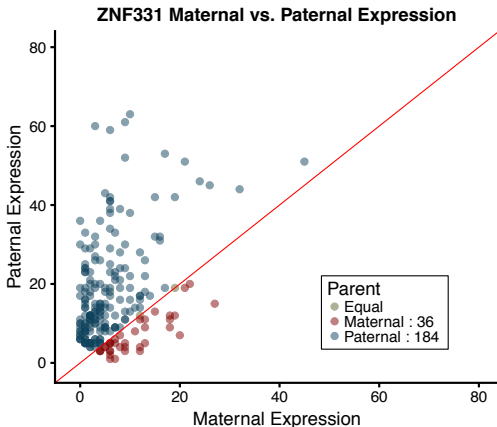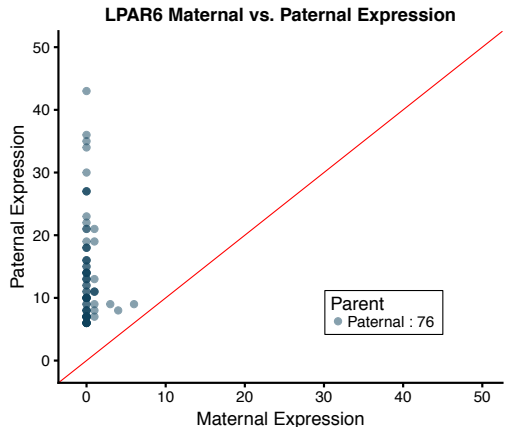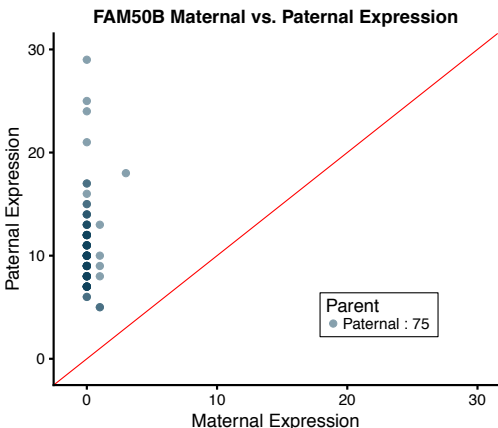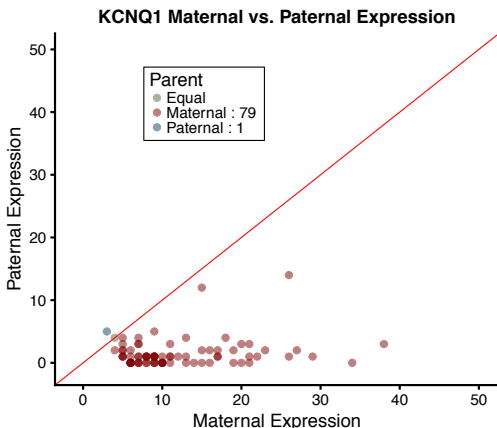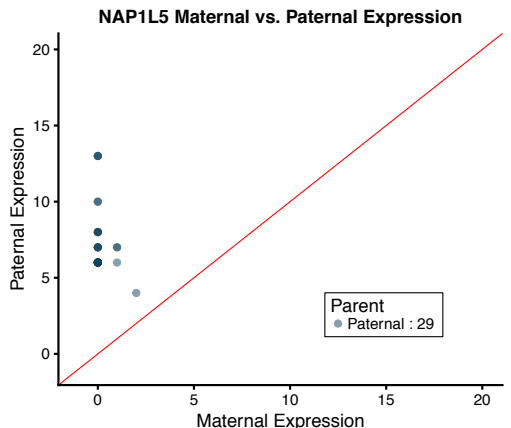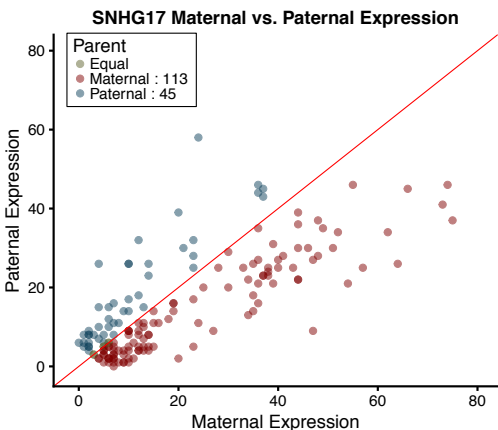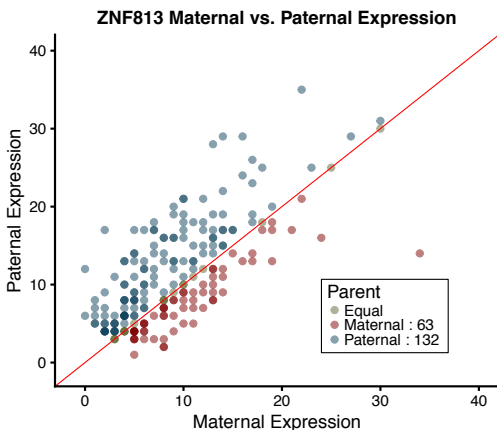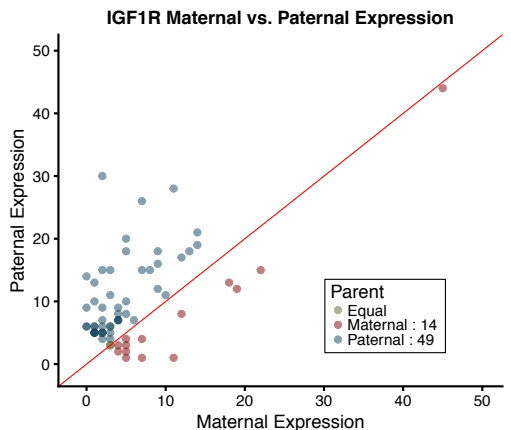

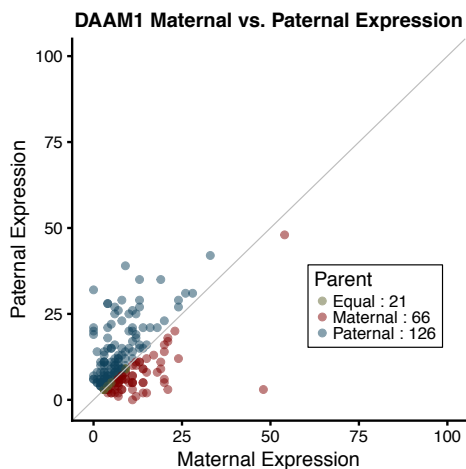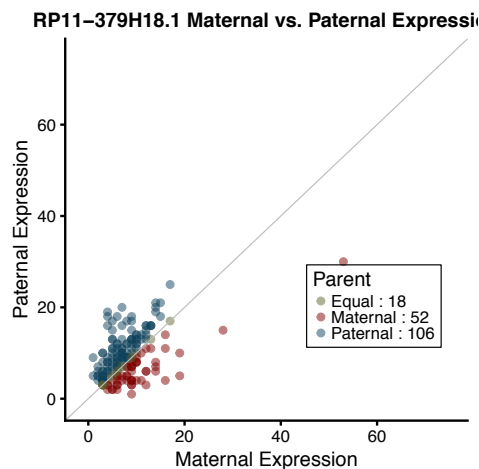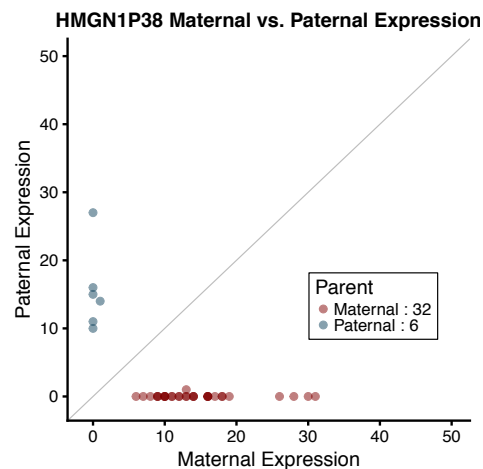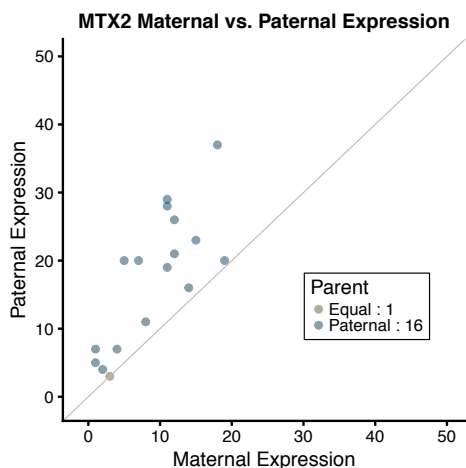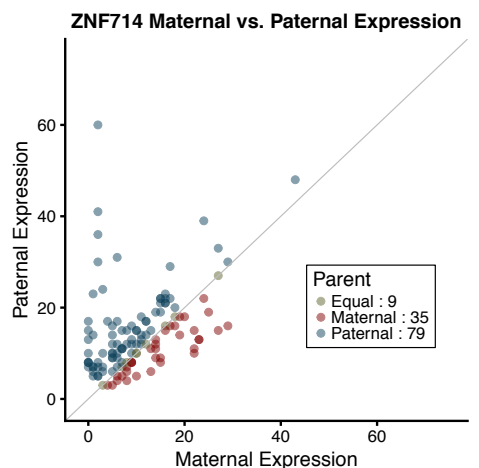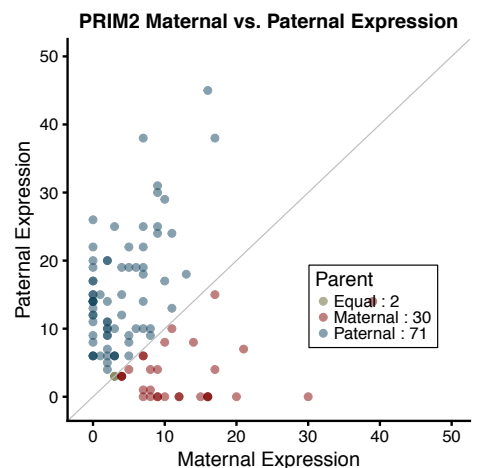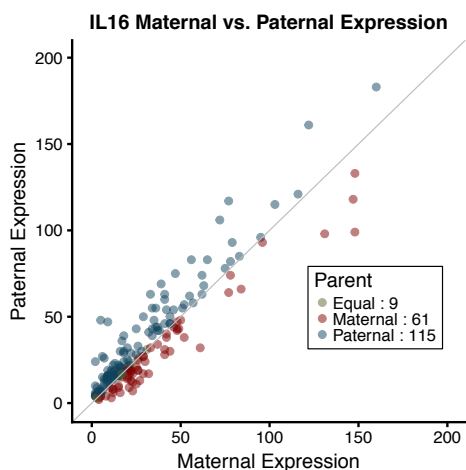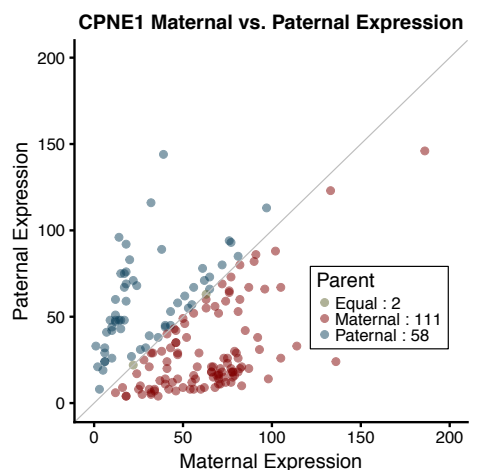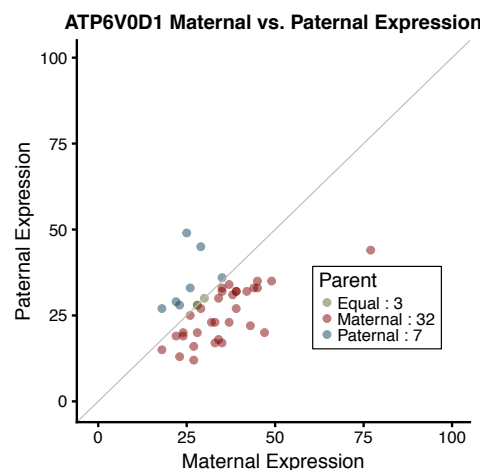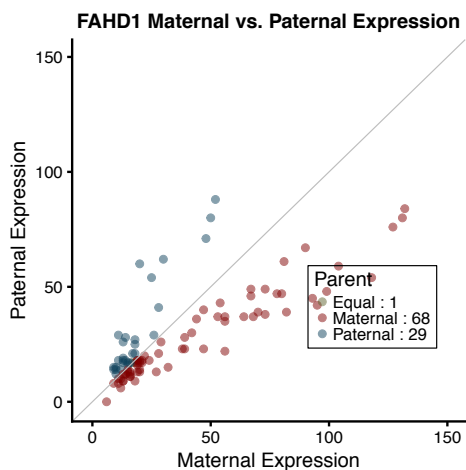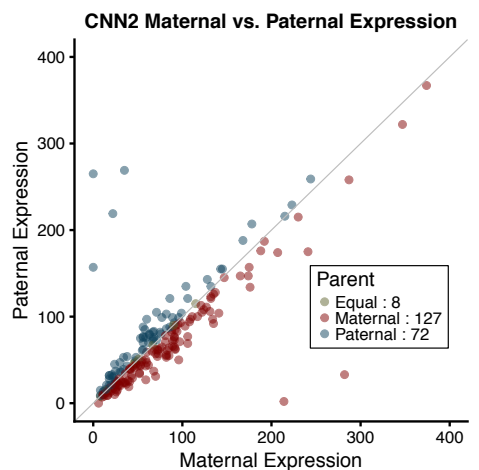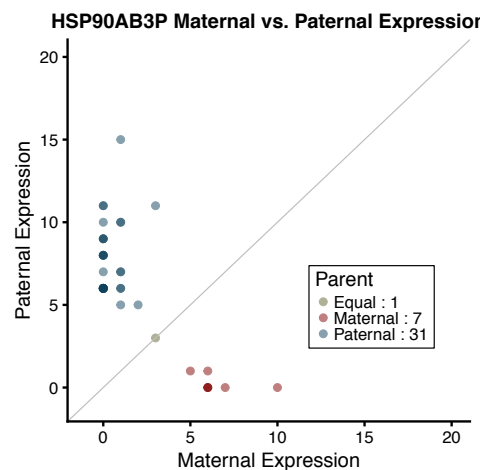

Supplement: S1 Fig — Plots of maternal (x-axis) and paternal (y-axis) gene expression for remaining genes with parent of origin asymmetry. (PDF) [file pone.0203906.s003.pdf]

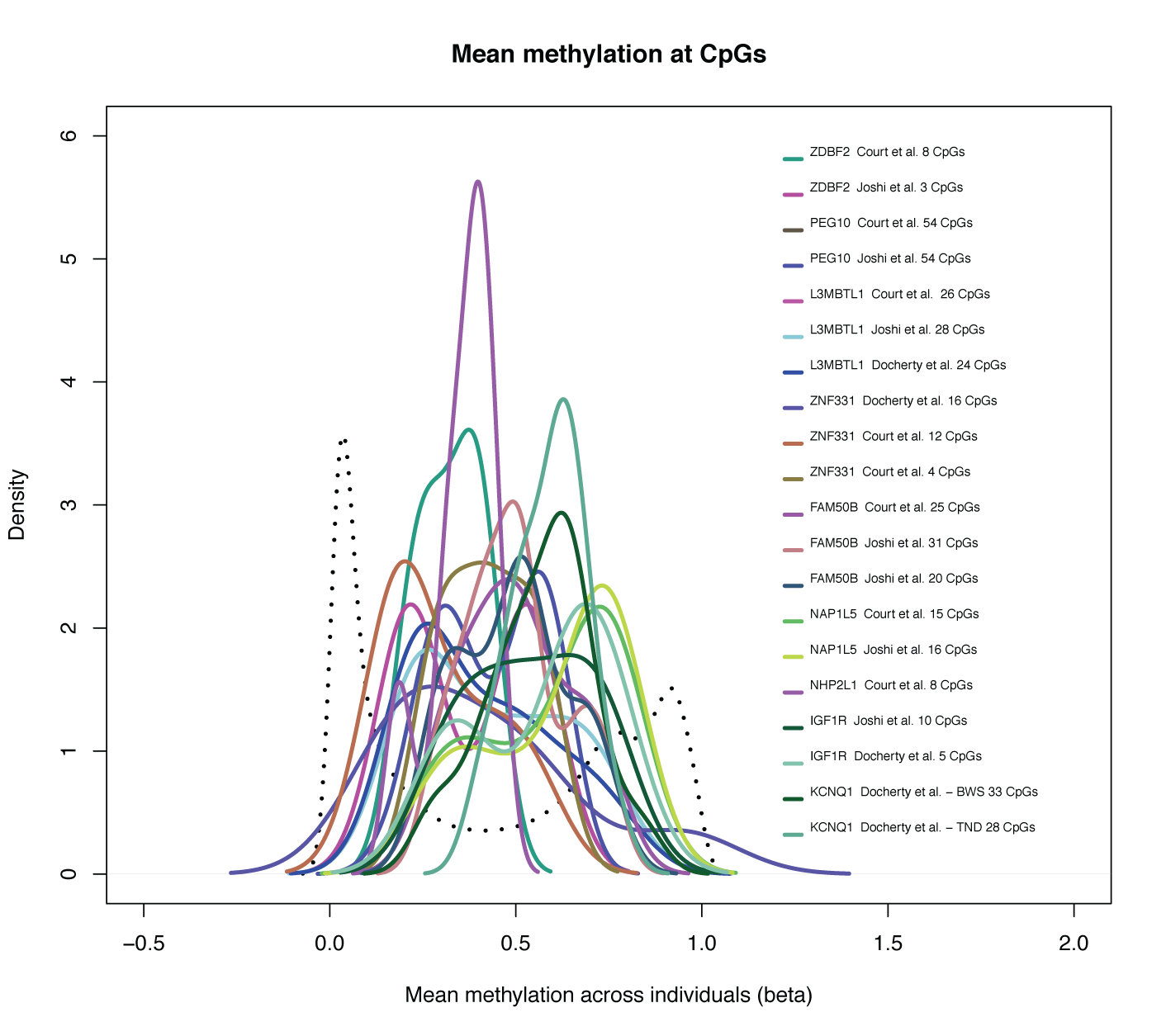

Supplement: S2 Fig — Density plot for Differentially Methylated Regions (DMRs) for imprinted genes from Joshi et al and Court et al. with beta values on the x-axis. (TIF) [file pone.0203906.s004.tif]

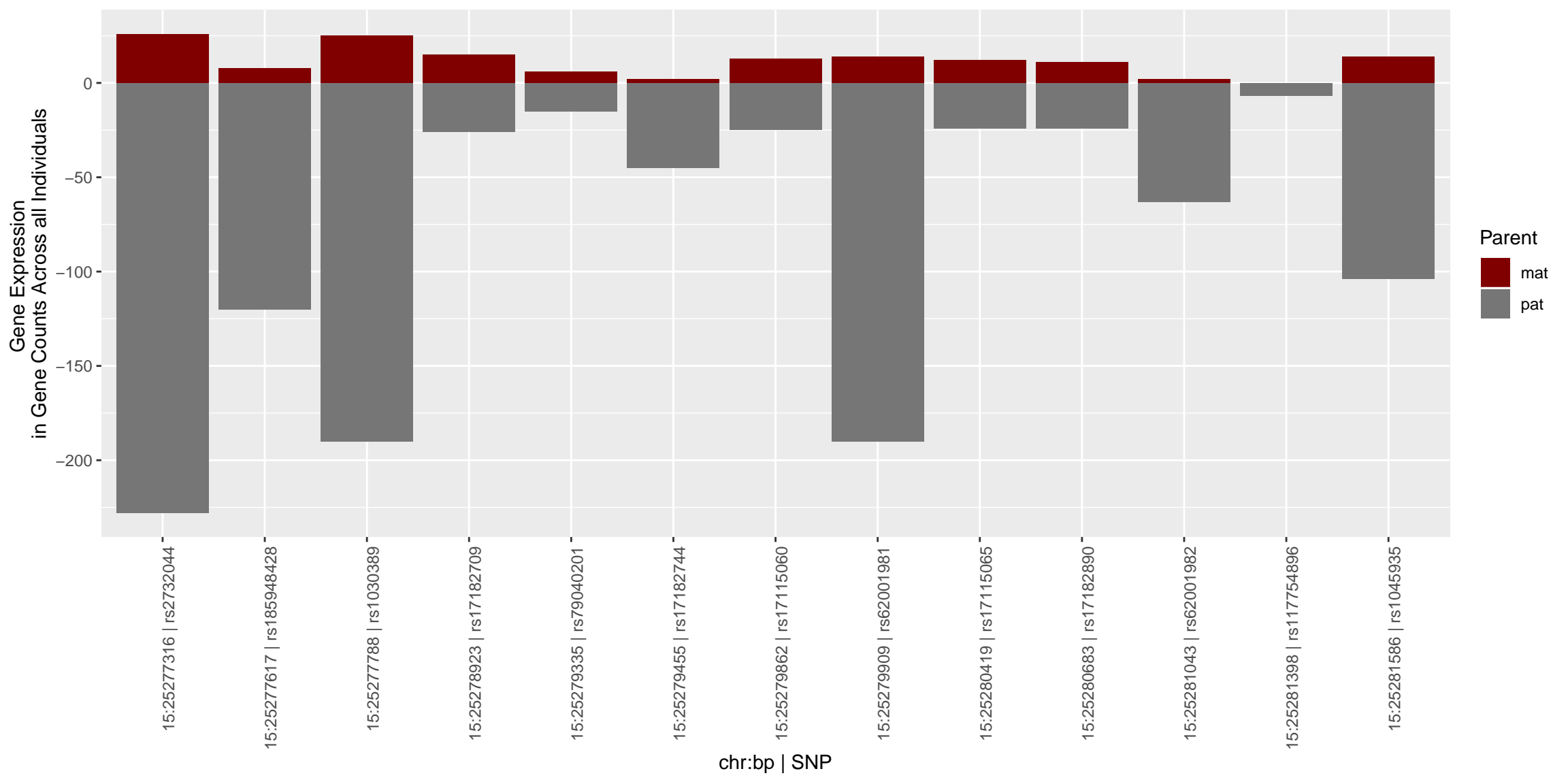

Supplement: S3 Fig — Number of reads used to assign reads to a parent across all the SNPs in gene PWAR6. SNPs are along the x-axis with chromosome and base pair location. The y-axis is gene expression in gene counts across all individuals. The grey is paternal expression and is negative on the y-axis. The red is maternal expression and is positive on the y-axis. (PDF) [file pone.0203906.s005.pdf]

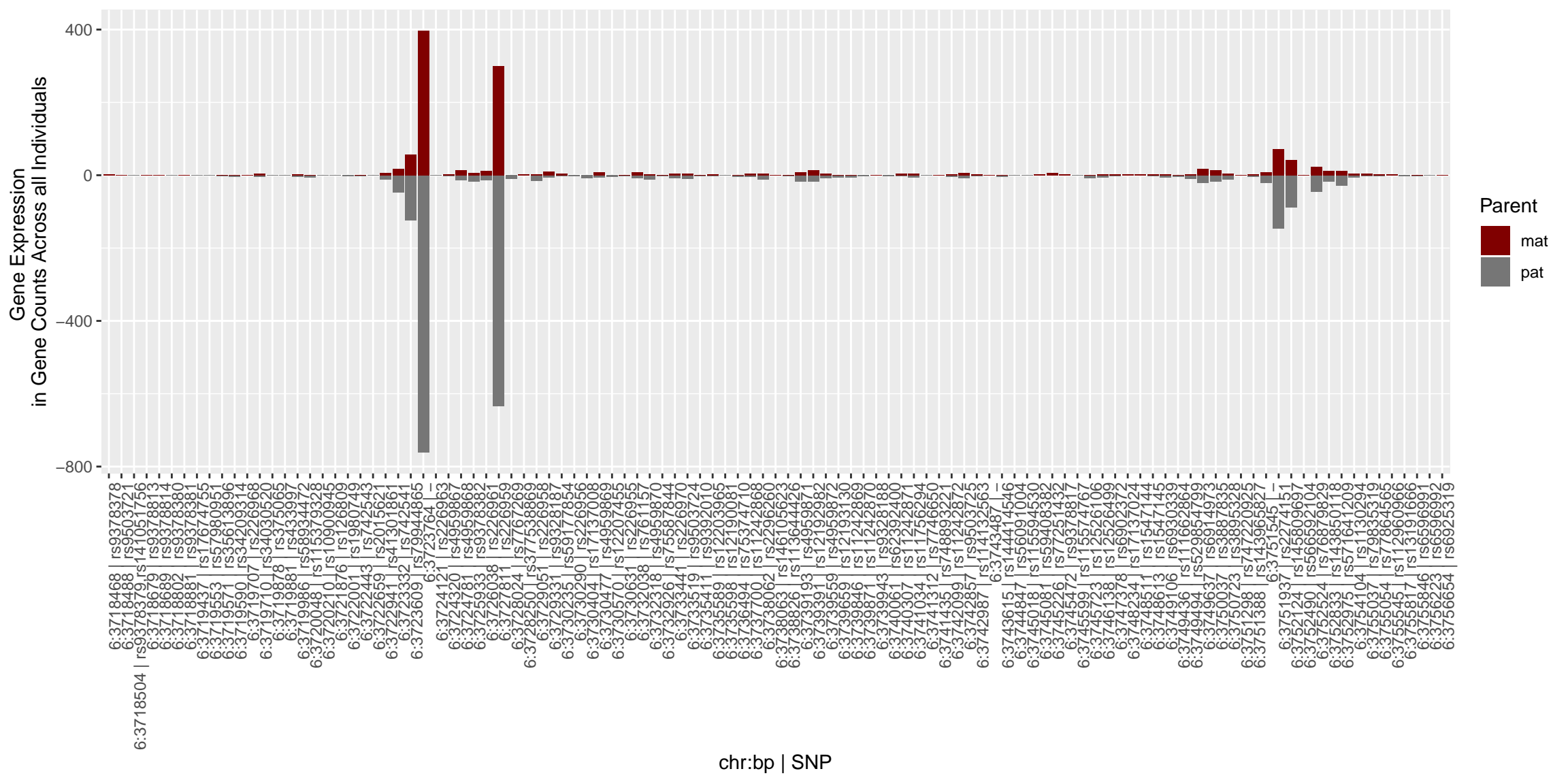

Supplement: S4 Fig — Number of reads used to assign reads to a parent across all the SNPs in gene PXDC1. SNPs are along the x-axis with chromosome and base pair location. The y-axis is gene expression in gene counts across all individuals. The grey is paternal expression and is negative on the y-axis. The red is maternal expression and is positive on the y-axis. (PDF) [file pone.0203906.s006.pdf]
